# Glycolysis and oxidative phosphorylation are essential for purinergic receptor-mediated angiogenic responses in vasa vasorum endothelial cells

**DOI:** 10.1152/ajpcell.00250.2016

**Published:** 2016-11-16

**Authors:** Martin Lapel, Philip Weston, Derek Strassheim, Vijaya Karoor, Nana Burns, Taras Lyubchenko, Petr Paucek, Kurt R. Stenmark, Evgenia V. Gerasimovskaya

**Affiliations:** ^1^Department of Pediatrics, University of Colorado Denver, Aurora, Colorado;; ^2^Department of Medicine, University of Colorado Denver, Aurora, Colorado; and; ^3^Department of Pharmacology, University of Colorado Denver, Aurora, Colorado

**Keywords:** vasa vasorum, endothelial cells, angiogenesis, purinergic receptors, cellular metabolism, glycolysis, OXPHOS

## Abstract

Angiogenesis is an energy-demanding process; however, the role of cellular energy pathways and their regulation by extracellular stimuli, especially extracellular nucleotides, remain largely unexplored. Using metabolic inhibitors of glycolysis (2-deoxyglucose) and oxidative phosphorylation (OXPHOS) (oligomycin, rotenone, and FCCP), we demonstrate that glycolysis and OXPHOS are both essential for angiogenic responses of vasa vasorum endothelial cell (VVEC). Treatment with P2R agonists, ATP, and 2-methylthioadenosine diphosphate trisodium salt (MeSADP), but not P1 receptor agonist, adenosine, increased glycolytic activity in VVEC (measured by extracellular acidification rate and lactate production). Stimulation of glycolysis was accompanied by increased levels of phospho-phosphofructokinase B3, hexokinase (HK), and GLUT-1, but not lactate dehydrogenase. Moreover, extracellular ATP and MeSADP, and to a lesser extent adenosine, increased basal and maximal oxygen consumption rates in VVEC. These effects were potentiated when the cells were cultured in 20 mM galactose and 5 mM glucose compared with 25 mM glucose. Treatment with P2R agonists decreased phosphorylation of pyruvate dehydrogenase (PDH)-E1α and increased succinate dehydrogenase (SDH), cytochrome oxidase IV, and β-subunit of F_1_F_0_ ATP synthase expression. In addition, P2R stimulation transiently elevated mitochondrial Ca^2+^ concentration, implying involvement of mitochondria in VVEC angiogenic activation. We also demonstrated a critical role of phosphatidylinositol 3-kinase and Akt pathways in lactate production, PDH-E1α phosphorylation, and the expression of HK, SDH, and GLUT-1 in ATP-stimulated VVEC. Together, our findings suggest that purinergic and metabolic regulation of VVEC energy pathways is essential for VV angiogenesis and may contribute to pathologic vascular remodeling in pulmonary hypertension.

angiogenesis, a process of new vessel formation, occurs in a variety of physiological and pathological conditions where hypoxia, ischemia, and inflammation are present (4, 14). The vasa vasorum (VV) is a microcirculatory network that provides oxygen and nutrients to the adventitia and media of large blood vessels and plays an active role in the vascular remodeling through angiogenesis and vasculogenesis (50, 58, 69). Previous studies have revealed a marked expansion of the VV network in the adventitia of the pulmonary artery (PA) from chronically hypoxic hypertensive calves and humans, implicating involvement of VV in pathological vascular remodeling in pulmonary hypertension (PH) (21, 49, 69). It is being recognized that hypoxia- and tumor-associated angiogenesis requires coordinated changes of intracellular signaling and cellular energy pathways (27, 37, 55). Yet, at present, the regulation of endothelial cellular energy pathways by angiogenic stimuli, and whether it results in metabolic reprograming of endothelial cell (EC) phenotype, remain largely unexplored.

Studies from several models suggest that differentiated EC represent a mostly glycolytic phenotype with relatively few mitochondria, denoting oxidative phosphorylation (OXPHOS) as the nonprimary source of ATP generation in EC (27, 76). It has been demonstrated that, similar to highly proliferative cancer cells, EC increase their glycolytic flux in response to angiogenic activation (36, 72). The induction of aerobic glycolysis (Warburg effect) was shown in EC proliferating under hypoxic conditions, thereby directing cellular energy metabolism to vascular remodeling (26, 51, 72, 82). Reliance on aerobic glycolysis was observed in proliferative PA lung microvascular EC (52) and in tip EC undergoing filopodia formation during vessel sprouting (22, 23). It has also been reported that PA EC from patients with idiopathic PH exhibit an abnormal metabolic phenotype, characterized by a metabolic switch to glycolysis accompanying reduced numbers of mitochondria (82).

Meanwhile, increasing data demonstrate that some highly proliferative tumor cells exhibit great diversity of energy utilization in growth responses and may retain the OXPHOS metabolism during oncogenic transformation (40, 65). In addition, cardiomyocytes, mesangioblasts, and inflammatory and stem cells are bivalent in their energy production and may undergo a switch from glycolysis to mitochondrial respiration during differentiation (53, 56, 62, 68). Even though EC are considered a “glycolytic” cell type, recent data also suggest that mitochondrial function is important in homeostatic regulation and angiogenic capacity of endothelium (20, 25, 64). Perinuclear clustering of mitochondria in lung EC has been demonstrated to contribute to ROS-dependent VEGF production (5). There is also evidence that proliferating EC increasingly depend on mitochondrial OXPHOS and, in particular, on the mitochondrial proton gradient (18). Together, these observations suggest the possibility that, depending on the activation and/or differentiation status, EC can undergo phenotypic and metabolic reprogramming. The relative contribution of endothelial glycolysis and OXPHOS to angiogenesis remains under investigated.

Regulation of angiogenesis via receptor-mediated signaling pathways has been fairly well studied. In contrast, the control of metabolism by receptor-mediated signaling has not, although this concept has been represented in several reports (28, 44, 72). Previously, our laboratory demonstrated that ATP can be released by VVEC in response to hypoxia and play a critical role in an autocrine/paracrine regulation of hypoxia-induced VV angiogenesis (80). However, regulation of cellular energy pathways via purinergic receptor-mediated signaling and whether it results in EC metabolic reprograming remains to be determined. In our present study, we used VVEC isolated from the vascularized areas of PA adventitia of chronically hypoxic calves that represent disease-relevant cell model to investigate signaling and metabolic responses involved in angiogenesis. Our data show that glycolysis and OXPHOS are both essential for VVEC angiogenesis and demonstrate a distinct role of P2 purinergic receptor (P2R) and P1 purinergic receptor (P1R) agonists in stimulating VVEC angiogenic responses, glycolysis, and OXPHOS. We also demonstrate a role of the phosphatidylinositol 3-kinase (PI3K)/Akt pathway in the regulation of several glycolytic and OXPHOS enzymes, as well as the facilitative glucose transporter GLUT-1. Based on results from our study, we speculate that regulation of VV angiogenesis by metabolic pathways and purinergic receptor agonists may contribute to pathological vascular remodeling in PH.

## METHODS

### 

#### VVEC culture.

VVEC were isolated from vascularized areas of PA adventitia of chronically hypoxic (14 days exposed to hypobaric hypoxia; barometric pressure = 430 mmHg) male Holstein calves, as previously described (30). Standard veterinary care was used following institutional guidelines, and the procedure was approved by the Institutional Animal Care and Use Committee (Department of Physiology, School of Veterinary Medicine, Colorado State University, Ft. Collins, CO). VVEC were isolated from PA adventitia, according to our laboratory's previously published methods (30) and cultured in DMEM supplemented with 10% fetal bovine serum (FBS) and endothelial growth supplement (Upstate Biotechnology, Charlottesville, VA). All studies were performed on cells between *passages 3* and *7*. Under these conditions, cells sustained consistent functional, morphological, and phenotypic characteristics.

#### DNA synthesis and proliferation assays.

To determine the rate of DNA synthesis, VVEC were plated in 24-well plates at a density of 1.2 × 10^4^ cells/well in DMEM supplemented with 10% FBS. On the next day, cells were rinsed with phosphate-buffered saline (PBS), and incubated in DMEM without serum for 72 h. Cells were preincubated with 2-deoxyglucose (2-DG; 2 mM), oligomycin (100 ng/ml), rotenone (0.1 μM), or FCCP (5 μM) for 20 min and stimulated with ATP, 2-methylthioadenosine diphosphate trisodium salt (MeSADP), or adenosine (100 μM each) in the presence of 0.125 μCi of [*methyl*-^3^H]thymidine (NEN Life Science Products, Boston, MA) for 24 h. Incorporated radioactivity was measured using a β-counter, as previously described (30). To determine the effects of metabolic inhibitors on VVEC proliferation, cells were plated in 96-well plates at a density of 1.2 × 10^3^ cells/well in DMEM supplemented with 1% FBS and grew in the presence of indicated metabolic inhibitors. Incubation media with all indicated components were changed each second day, and cell proliferation rate was assessed using CyQuant proliferation kit (Invitrogen, Carlsbad, CA), according to the manufacturer's instructions. Fluorescence intensity was determined using a plate reader (BMG Labtech).

#### Cell migration.

To assess the effects of metabolic inhibitors on angiogenic activity of VVEC, migration and tube formation in vitro assays were performed as previously described (30). Growth arrested cells (DMEM without serum, 72 h) were seeded on top of the transwell-permeable support of the Boyden chamber (8-mm pore size; Corning) at the amount of 1 × 10^5^ cells and preincubated with oligomycin (100 ng/ml), rotenone (0.1 μM), 2-DG (2 mM), and FCCP (5 μM) for 20 min. ATP, MeSADP, or adenosine (500 μM each) was added to the lower compartment to initiate migration. After 24 h, nonmigrated cells were scraped off from the top of the filters; migrated cells were fixed in methanol for 15 min at room temperature and stained with 0.2% crystal violet in 2% ethanol for 15 min. Migrated cells were photographed under ×20 magnifications in a phase-contrast microscope (Nikon) at three random fields/well and counted using ImageJ software.

#### Tube formation.

Tube formation assay was carried out in ibidi angiogenesis slides (ibidi USA, Madison, WI) precoated with 10 μl of Growth Factor Reduced Matrigel Matrix (BD Bioscience). Growth-arrested VVEC were seeded on polymerized Matrigel in triplicate at a density of 1.7 × 10^4^ cells/well and preincubated with oligomycin (100 ng/ml), rotenone (0.1 μM), 2-DG (2 mM), and FCCP (5 μM) for 20 min before stimulation with ATP, MeSADP, or adenosine (100 μM each). Images were captured after 6–8 h using a digital camera connected to a phase-contrast microscope (Nikon) at three to five random fields. The number of tubes and average tube length were determined using ImageJ64 analysis.

#### Cell culture for bioenergetic analysis.

VVEC were split into two 60-mm petri dishes containing complete growth media (DMEM, 10% FBS) for 1 h to allow for cell attachment and changed to growth medium containing 25 mM glucose or 20 mM galactose + 5 mM glucose thereafter. Cells were maintained for 36 h at 37°C and 5% CO_2_ until 90–95% confluence was reached. On reaching optimal confluence, cells were trypsinized and seeded in Seahorse XF 24-well cell culture plates (Seahorse Bioscience, Billerica, MA) at 2.0 × 10^4^ cells/well in HBSS in the absence of sodium bicarbonate, calcium, and magnesium (Corning, One Riverfront Plaza, NY). Seeding was performed using a two-step process in an initial seeding volume of 200 μl/well of respective growth media, followed by a 1-h incubation to allow attachment of cells. An additional 100 μl of growth media were added following 1-h incubation. Cells were then placed back into incubators for 24 h before serum starvation. Media in XF24 plates were changed to 500-μl respective DMEM without serum 24 h after initial incubation. Cells were placed back into incubation for 36 h of serum starvation. Before metabolic flux experiment, unbuffered Seahorse XF Assay medium with 25 mM glucose and 1 mM sodium pyruvate adjusted to pH 7.4 was prepared. Alternatively, before glycolysis stress test assay experiments, unbuffered Seahorse XF Base medium containing 2 mM glutamine and no glucose nor sodium pyruvate adjusted to pH 7.4 was prepared and used as assay medium. Cells were removed from incubation, and DMEM media was changed to assay medium. Cells were then incubated at 37°C in the absence of CO_2_ for a total of 1 h before metabolic flux analysis was performed.

#### Bioenergetic analysis via extracellular flux measurements.

Using a Seahorse Bioscience XF24 Extracellular Flux Analyzer, simultaneous measurements of oxygen consumption rate (OCR) and extracellular acidification rate (ECAR) were obtained from intact cells to infer dynamic cellular energy metabolism state in response to nucleotides, as well as short- and long-term growth medium treatments. Injector compounds were prepared the day of experiment in XF Assay Medium containing 0.8 mM Mg^2+^, 1.8 mM Ca^2+^, 143 mM NaCl, 5.4 mM KCl, 0.91 mM NaH_2_PO_4_, 2 mM l-Ala-Gln (Glutamax), and 3 mg/l phenol red, or XF Base Medium containing 0.8 mM Mg^2+^, 1.8 mM Ca^2+^, 143 mM NaCl, 5.4 mM KCl, 0.91 mM NaH_2_PO_4_, and 3 mg/l phenol red (Seahorse Bioscience). Nucleotides to be injected (ATP, MeSADP) and adenosine were obtained from Sigma Aldrich (St. Louis, MO). All experimental compounds were prepared in respective experimental assay media, wherein glucose, nucleotides, oligomycin, and 2-DG were prepared in pH-adjusted media using XF Base media, and nucleotides, oligomycin, FCCP, rotenone, and antimycin A were prepared in pH-adjusted medium using XF Assay medium, as described previously for glycolysis and mitostress test assays, respectively. Experiments designed to test initial nucleotide responses utilized port A injectors to introduce nucleotides, followed by respective data point collection, based on the protocol for the parameter of interest. Experiments designed to test nucleotide response post-glycolytic activation by glucose utilized port B injectors. All subsequent compounds were assigned traditional injector ports, as detailed in manufacturer assay protocols. For both mitostress and glycolytic stress test assays, three baseline measurements were obtained using a three-command loop design entailing 1-min mixing, 2-min delay, and 3-min measurement commands. Initial baseline measurements were followed by injection of port A compounds and either nine-command loops using 1-min mixing, 2.5-min delay, and 3-min measurement settings for mitostress assays, or three-command loops using 1-min mixing, 2-min delay, and 3-min measurement settings for glycolysis stress test assays. Both mitostress test assays and glycolysis stress test assays were then followed by injection of B ports using respective three-command loop mixing, delay and measurement settings, and then injection of C ports, and so on. Data collected by Seahorse Extracellular Flux Analyzer was then transferred to Excel format for data analysis employing Graphpad Prism for graphical representation.

#### Western blot analysis.

Growth arrested VVEC (DMEM without serum, 72 h) were stimulated with ATP, MeSADP, or Ado (100 μM) for 30 min, 4 h, and 24 h. When PI3K inhibitor (LY294002) or Akt inhibitor (GSK690693) were tested, cells were preincubated with the indicated compounds for 20 min before the stimulation. At the end of incubation, cells were washed with cold PBS and lysed on ice for 30 min in buffer containing 20 mM Tris·HCl (pH 7.5), 150 mM NaCl, 1 mM EDTA, 10% glycerol, 1% Nonidet P-40, 1% Triton, 1 mM dithiothreitol, 10 mM glycerophosphate, 10 mM NaF, 1 mM sodium orthovanadate, 10 μg/ml leupeptin, 10 μg/ml aprotinin, 10 μg/ml pepstatin A, and 1 mM phenylmethylsulfonyl fluoride. Cellular debris was eliminated by centrifugation for 10 min at 12,000 *g*. Equivalent amounts of total cell protein (20 μg) were subjected to SDS-PAGE and transferred onto nitrocellulose membranes (GE Health Care, Pittsburgh, PA). Membranes were blocked in PBS containing 0.1% Tween 20 and 1% BSA for 1 h at room temperature and incubated with primary antibodies overnight at 4°C, and with secondary antibodies for 1 h at room temperature. The following primary antibodies were used: phospho-Akt, Akt, hexokinase (HK) 2 (1:1,000, Cell Signaling Technology, Danvers, MA); phospho-lactate dehydrogenase (LDH) (1:500; Cell Signaling Technology), LDH (1:1,000; Abcam, Cambridge, MA); pyruvate dehydrogenase (PDH), phospho-PDH (1:2,000; Abcam); phospho-PFKB3 (1:000; Aviva Systems Biology, San Diego, CA), PFKB3 (1:2,000; Abcam); succinate dehydrogenase (SDH) (1:1,000; Abcam); cytochrome oxidase subunit IV (COX IV) and F_1_F_0_ ATP synthase β-subunit (1:000; Santa Cruz Biotechnology, Santa Cruz, CA), and GLUT-1 (1:1,000; Abcam). All antibodies were diluted in PBS containing 0.1% Tween 20 and 1% BSA. Immunoreactive bands were detected using ECL kit (Renaissance, NEN Life Science Product), followed by exposure to Optimum X-ray Film (Life Science Products, Frederick, CO). In all experiments, equal sample loading and transfer were verified by staining nitrocellulose membranes with Ponceau solution and by probing with anti-GAPDH antibodies (dilution 1:1,000; Cell Signaling Technology). Calculations of the protein band densities were performed using ImageJ software.

#### GLUT-1 immunofluorescence.

VVEC were plated on eight-well chamber slides (Labtek, Grand Rapids, MI), serum starved, and stimulated with ATP (100 μM) for 0.5–24 h. Stimulated cells were washed three times with PBS (5 min each) and blocked in PBS containing 5% normal goat serum and 0.3% Triton X for 1 h at room temperature. Cells were incubated with rabbit polyclonal antibody against GLUT-1 (Abcam no. 115730; 1:400) overnight at 4°C. After the incubation, cells were washed as indicated above and incubated with fluorescein-conjugated *Grifonia Simplicifolia* lectin (Vector Labs, Burlingame, CA; no. FL-1101) at the concentration of 2 μg/ml for 1 h at room temperature. Following this step, cells were washed and incubated with the secondary, goat anti-rabbit antibody Alexa Fluor-594 (Jackson Labs, Farmington, CT; no. 111-585-003; 1:400), for 1 h at room temperature. Finally, cells were washed, air dry in the dark, and treated with Prolong Gold anti-fade with 4,6-diamidino-2-phenylindole (Cell Signaling Technology, no. 8961-I). Images were captured using EVOS imaging system under ×40 magnification.

#### Measurement of lactate and ATP production.

Cells were grown and stimulated as described above for Western blot analysis. Aliquots of total cell lysates (30 μl) were analyzed with l-Lactate Assay Kit (Cayman Chemical, Ann Arbor, MI). For ATP measurements, 300 μl of conditioned media were collected into chilled polypropylene tubes (Sigma, St. Louis, MO) and centrifuged at 12,000 *g* for 10 min to remove any cell debris. Media were frozen at −80°C for subsequent measurement. ATP sample concentrations were detected with the luciferase luciferin kit (ENLITEN ATP Assay System, Promega, Madison, WI). Both lactate and ATP measurements were performed using a Polarstar Omega microplate reader (BMG Labtech, Cary, NC). The samples were compared with a lactate or ATP or standard curve performed in each individual experiment.

#### Intracellular Ca^2+^ measurements.

VVEC were cultured in glass-bottom dishes suitable for fluorescent imaging (MatTek, Ashland, MA) and growth-arrested in serum-free DMEM for 72 h before the experiments. Immediately before the Ca^2+^ measurement, cells were incubated in the presence of membrane-permeable Ca^2+^ indicator rhodamine 2AM (Invitrogen R-1245MP; red color in Fig. 7*A*) at 1 μM for 30 min at room temperature, washed with media, and left to recover for 10 min to reduce the spontaneous intrinsic Ca^2+^ activity. In some experiments, cells were pretreated with the inhibitor of Ca^2+^ transport to mitochondria, ruthenium red (1 μM, 30 min). Time-lapse image acquisition was started, and, after establishing the baseline for 150 s, the acquisition was paused to perfuse the cell culture dish with media containing 100 μM ATP. Image acquisition was resumed immediately after the perfusion. To determine Ca^2+^ signal localization to mitochondria, Ca^2+^ influx was assayed in live cells coloaded with MitoTracker Deep Red FM (Invitrogen M22426) at 0.8 μM for 30 min at 37°C and with rhodamine 2AM (red and green, respectively, in Fig. 7*B*). In both experimental settings, the time-lapse images were captured with 10-s intervals on Nikon TE2000 microscope equipped with Cooke CCD SensiCam. SlideBook software was used for image acquisition and analysis. Averaged single-cell Ca^2+^ traces were measured by fluorescence intensity levels within hand-drawn regions of interest in individual cells (*n* = 15–20).

#### Statistical analysis.

For the analysis of variances between groups of data, two-way ANOVA with a Bonferroni posttest was performed using GraphPad Prism 4.0. Data are expressed as means ± SE. A *P* value of <0.05 was considered statistically significant. Three to five individual experiments were carried out for each assay. VVEC populations were isolated from at least three different animals.

## RESULTS

### 

#### VVEC growth and angiogenesis requires both glycolysis and OXPHOS.

**Fig. 1.**
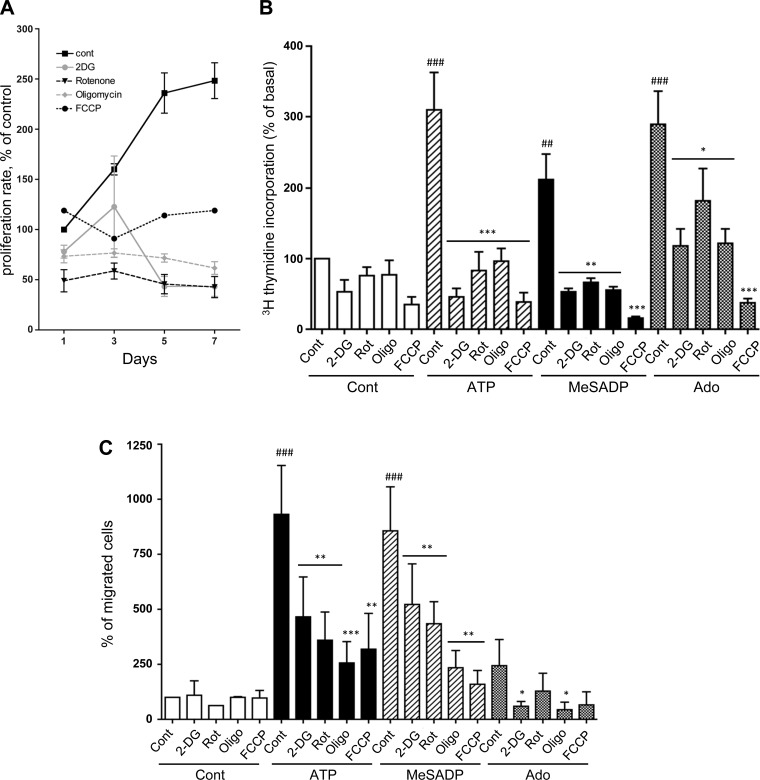
Glycolysis and mitochondrial oxidative phosphorylation are necessary for VVEC proliferative and migratory responses. *A*: proliferation rates were determined in VVEC grown in DMEM/1% FBS in the presence of oligomycin (Oligo; 10 ng/ml), rotenone (Rot; 0.1 μM), 2-deoxyglucose (2-DG; 0.2 mM), and FCCP (0.5 μM) The media with indicated inhibitors were refreshed each second day. Cell proliferation rate was assessed using a fluorescent CyQuant proliferation kit. Values are means ± SE from 3 independent experiments. *B*: [^3^H]thymidine incorporation was determined in growth-arrested VVEC (DMEM without serum, 72 h) in response to stimulation with extracellular ATP (100 μM). Cells remained untreated or were treated with 2-DG (2 mM), Rot (0.1 μM), Oligo (100 ng/ml), or FCCP (2 μM) 20 min before stimulation. Values are means ± SE from 3 independent experiments. ##*P* < 0.01. ###*P* < 0.001. *P < 0.05, **P < 0.01, and ***P < 0.001 compared with ATP-treated cells. *C*: for migration assay, growth-arrested cells were seeded in Boyden chamber-permeable inserts and pretreated with the indicated for 20 min. ATP, MeSADP, or adenosine (Ado; 500 μM) were added to the lower compartment to initiate migration. Number of migrated cells was evaluated as described in methods. Values are means ± SE from 4 independent experiments. ###*P* < 0.001, control vs. ATP-stimulated cells. *P < 0.05, **P < 0.01, and ***P < 0.01, untreated vs. treated with inhibitors.

To determine the role of glycolysis and OXPHOS in VVEC proliferative responses, we used pharmacological inhibitors that target different steps of cellular energy production. Our data show that treatment of VVEC with glycolytic inhibitor 2-DG (glucose analog that inhibits HK), and the inhibitors of mitochondrial complex I (rotenone) and complex V (oligomycin), significantly decreased cell proliferation rate (Fig. 1*A*). Following this observation, the effects of metabolic inhibitors were evaluated on purinergic receptor-mediated angiogenic responses. Extracellular ATP and the stable ADP analog MeSADP (both P2R agonists) were used in this study based on our laboratory's previously published data showing pro-angiogenic effect of these agonists in VVEC (30, 46, 80), whereas the effects of extracellular adenosine (P1R agonist) on angiogenic and metabolic responses have not been previously investigated. Treatment with ATP, MeSADP, and adenosine robustly increased VVEC DNA synthesis (Fig. 1*B*). 2-DG, rotenone, and oligomycin, as well as mitochondrial uncoupler, FCCP, significantly decreased the rate of DNA synthesis in cells exposed to all three purinergic receptor agonists. The more dramatic inhibitory effect was observed in response to 2-DG and FCCP compared with rotenone and oligomycin, under both basal and nucleotide-stimulated conditions.

Furthermore, a role of cellular energy pathways in VVEC angiogenesis was determined in cell migration and tube formation assays. As shown in Fig. 1*C*, ATP and MeSADP were almost equipotent in stimulating VVEC migration. In contrast, adenosine was not effective, indicating that P1Rs do not mediate the migration response. The responses to ATP and MeSADP were significantly reduced by 2-DG (by 30–50%) and rotenone (by 50–60%) and even more dramatically reduced by oligomycin (by 70–75%) and FCCP (66–72%).

**Fig. 2.**
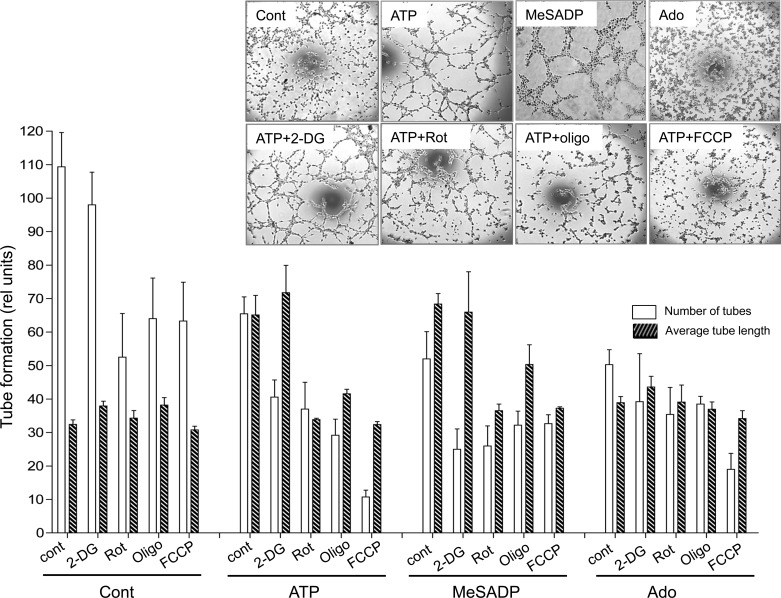
Effects of metabolic inhibitors on purinergic receptor-mediated VVEC tube formation. Growth-arrested VVEC were seeded in angiogenesis slides (ibidi) covered with polymerized Growth Factor Reduced Matrigel, as described in methods. Cells (1.7 × 10^4^ cells/well) were pretreated with 2-deoxyglucose (2-DG; 2 mM), rotenone (Rot; 0.1 μM), oligomycin (Oligo; 100 ng/ml), or FCCP (2 μM) for 20 min, followed by stimulation with ATP, MeSADP, or adenosine (Ado; 100 μM) for 6 h. Images were captured using a digital camera connected to a phase-contrast microscope (Nikon). *Top*: shown are representative images from one of five experiments performed on three VVEC populations. *Bottom*: tube formation responses were characterized by evaluation of number of tubes and average tube length, using ImageJ64 program. Values are means ± SE.

Tube formation assay on growth factor reduced Matrigel demonstrated VVEC rearrangement into tubular-like network in response to ATP and MeSADP, but not to adenosine (Fig. 2). The observed tube formation response was evaluated as increase in tube length and decrease in number of tubes in nucleotide-stimulated cell compared with control, either treated or untreated with inhibitors. Rotenone, oligomycin, and FCCP prevented tube formation; however, 2-DG did not show this effect. Similar inhibitory effects on tube formation were observed when cells were stimulated with MeSADP. Together, these studies demonstrated that both glycolysis and OXPHOS play a critical role in angiogenic responses in VVEC, and purinergic receptor agonists show diverse potency in mediating proliferation, migration, and tube formation.

#### P2Rs stimulate glycolysis in VVEC.

**Fig. 3.**
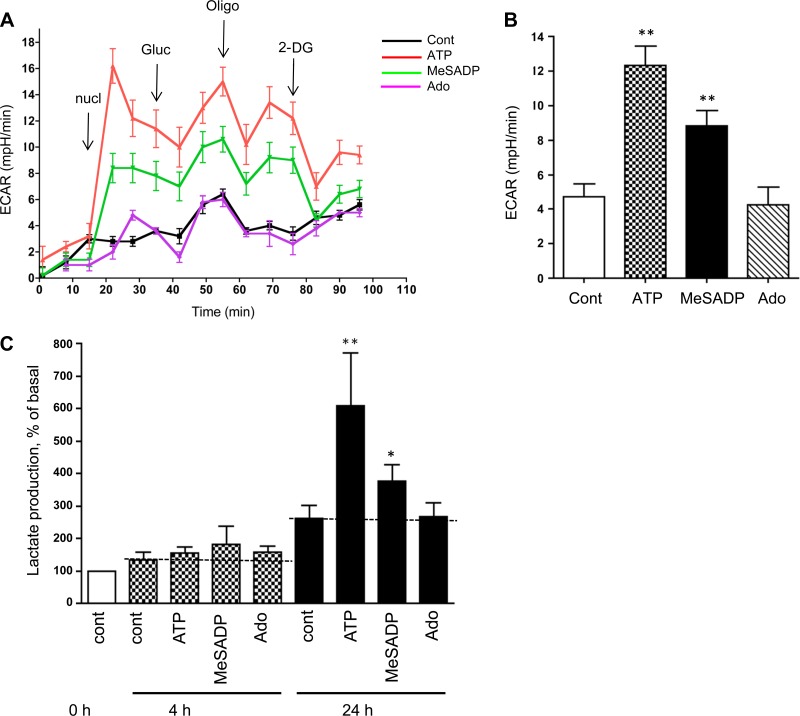
Extracellular nucleotides (nucl) ATP and MeSADP, but not adenosine (Ado), stimulate aerobic glycolysis in VVEC. *A*: glycolytic rate was measured as extracellular acidification rate (ECAR) using XF24 Extracellular Flux Analyzer (Seahorse Bioscience), as described in methods. ATP, MeSADP, or Ado (100 μM each), glucose (Gluc; 10 mM), oligomycin (Oligo; 0.8 μM), or 2-DG (10 mM) were injected at the indicated times. *B*: data show ECAR rates measured between 15 and 35 min of each agonist treatment. Values are means ± SE from 4 independent experiments. **P < 0.01, nonstimulated vs. agonist-stimulated cells. *C*: lactate was determined in a conditioned media of VVEC stimulated with ATP, MeSADP, or Ado for indicated times, as described in methods. The average basal lactate production level was 23.9 ± 3.3 μM. Values are means ± SE from 3–5 independent experiments. **P < 0.01 and *P < 0.05, nonstimulated vs. agonist-stimulated cells. Cont, control.

To examine if purinergic receptor stimulation activates VVEC glycolysis, we used the Seahorse Bioscience XF24 Extracellular Flux Analyzer, which measures glycolytic response as ECAR in intact cells and enables dynamic treatment of cells through injector ports. For the ECAR experiments, VVEC were cultured in a standard growth medium with 25 mM glucose and incubated with unbuffered Seahorse XF Base medium without glucose 1 h before metabolic flux measurements. Stimulation with extracellular ATP and MeSADP resulted in a dramatic increase in ECAR by 3.3- and 1.9-fold, respectively, in VVEC (Fig. 3, *A* and *B*). In contrast, stimulation with extracellular adenosine did not have any effect on ECAR. Injection of oligomycin resulted in a biphasic ECAR response (a decrease followed by an increase), but no additional increases above the nucleotide- and glucose-stimulated level were observed. The ECAR response was muted by the addition of glycolytic inhibitor 2-DG, and cells tended to maintain ECAR above baseline. To complement these data, we evaluated the effects of purinergic receptor agonists on lactate production. As shown in Fig. 3*C*, ATP and MeSADP stimulated lactate production by 2.4- and 1.45-fold, respectively. In contrast, adenosine was ineffective, indicating that P2R, but not P1R, are positive regulators of VVEC glycolysis.

**Fig. 4.**
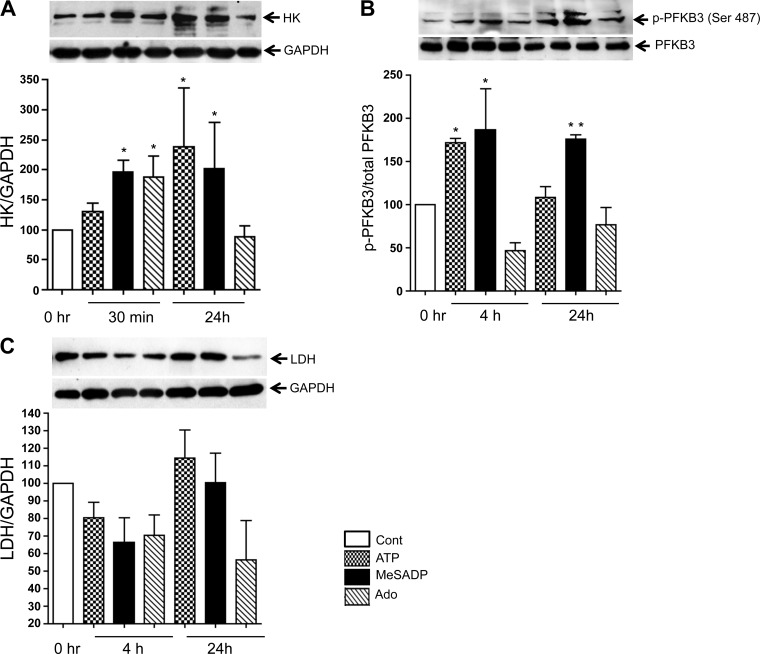
Extracellular ATP, MeSADP, and adenosine (Ado) upregulate key glycolytic enzymes in VVEC. Growth-arrested cells were stimulated with ATP, MeSADP, or Ado (100 μM each). HK, phospho-PFKB3, and lactate dehydrogenase (LDH) were determined in total cell lysates by Western blot analysis. Values are means ± SE from 3–5 independent experiments performed on 3 cell populations. **P < 0.01 and *P < 0.05, nonstimulated vs. agonist-stimulated cells.

The upregulation of glycolysis prompted us to examine the effects of purinergic receptor activation on the expression level and/or activation of key glycolytic enzymes. Stimulation with extracellular ATP, MeSADP, and, to a lesser degree, adenosine increased HK2 expression at 30 min of incubation. The effects of ATP and MeSADP, but not adenosine, were observed on HK2 expression level at 24 h of incubation (Fig. 4*A*). Under the same conditions, ATP increased the phospho-PFKB3 level at 4 h, and MeSADP exerted this effect at 4 and 24 h. Stimulation with adenosine did not increase phospho-PFKB3 level (Fig. 4*B*). We also found that ATP slightly increased the expression of LDH that was observed after 24 h of stimulation; however, MeSADP did not show this effect (Fig. 4*C*). Moreover, stimulation adenosine decreased the LDH level by about twofold compared with the basal level.

**Fig. 5.**
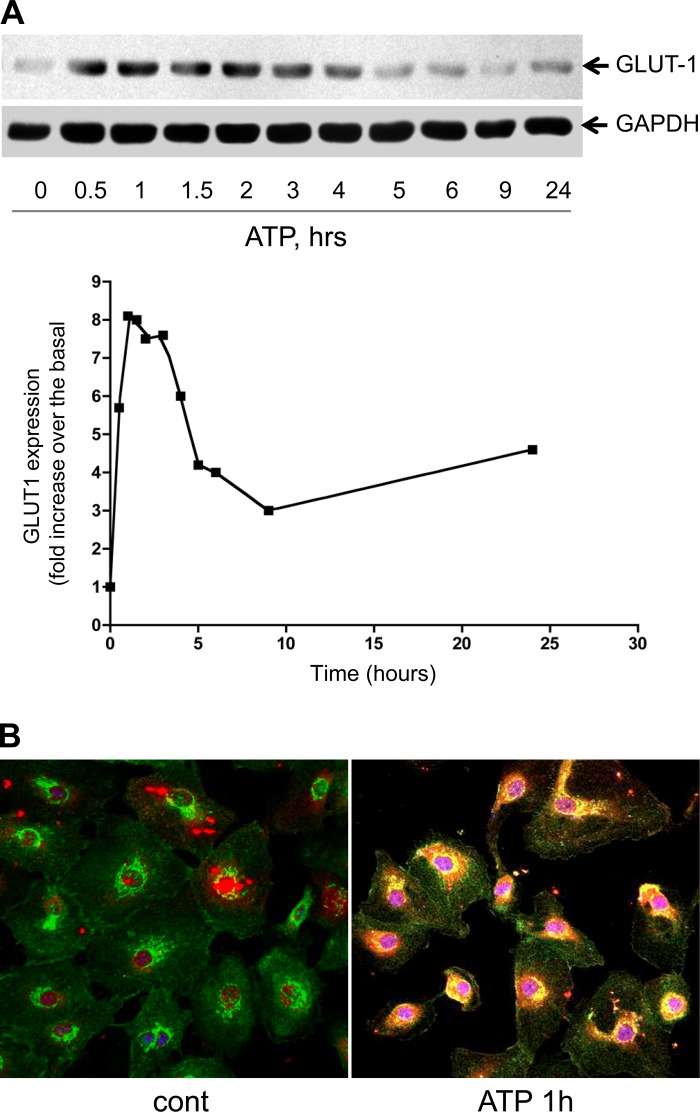
Extracellular ATP induces GLUT-1 expression and plasma membrane translocation. *A*: growth-arrested cells (DMEM without serum, 72 h) were stimulated with ATP for indicated times. GLUT-1 was determined in total cell lysates by Western blot analysis. *B*: immunofluorescent analysis of GLUT-1 expression and localization in ATP-stimulated VVEC. After 1 h of treatment, cells were fixed and incubated with fluorescein-conjugated *Grifonia Simplicifolia* lectin (2 μg/ml), followed by the incubation with rabbit polyclonal antibody against GLUT-1 (1:200), secondary goat anti-rabbit antibody conjugated to Alexa Fluor-594 (1:400), and Prolong Gold anti-fade with DAPI, as described in methods. Images were captured under ×40 magnification.

Considering that glucose is a primary energy source for cellular energy pathways, we also examined the effects of purinergic receptor agonists on GLUT-1 expression, a facilitative glucose transporter known to play a critical role in glucose uptake in EC (66). Using Western blot analysis (Fig. 5*A*), we found that stimulation with extracellular ATP (100 μM) significantly increased GLUT-1 expression, which was observed between 30 min and 4 h, with a maximal response at 1.5 h. MeSADP was as potent as ATP, but the effect of adenosine was less significant (data not shown). We also found that stimulation with extracellular ATP results in a GLUT-1 translocation to the plasma membrane. In these experiments, VVEC were simultaneously labeled with fluorescein-conjugated *Grifonia Simplicifolia* lectin and immunoprobed with anti-GLUT-1 antibodies (Fig. 5*B*).

#### OXPHOS in VVEC is regulated by purinergic receptors and galactose.

**Fig. 6.**
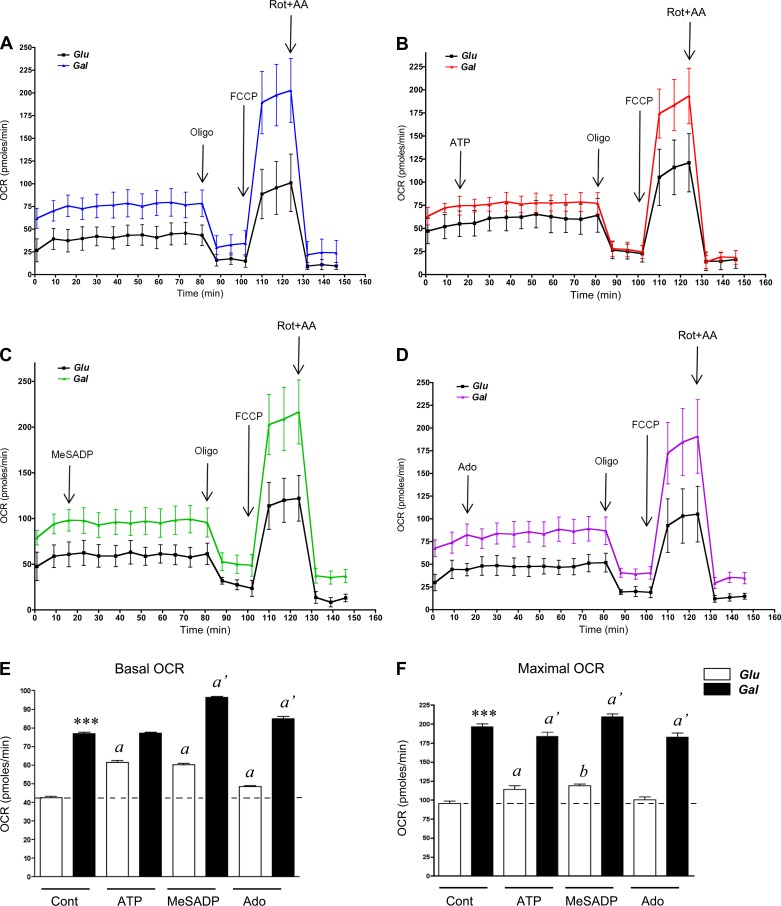
Extracellular nucleotides, adenosine (Ado), and galactose (Gal) increase respiratory capacity of VVEC. Mitochondrial respiration [oxygen consumption rate (OCR)] analysis was performed using XF24 Extracellular Flux Analyzer in VVEC cultured in media containing either 25 mM glucose (Glu) or 5 mM Glu and 20 mM Gal, as described in methods. *A–D*: control (*A*), ATP (*B*), MeSADP (*C*), Ado (*D*), oligomycin (Oligo; 0.8 μM), FCCP (2 μM), rotenone (Rot; 0.1 μM), and antimycin A (AA; 2 μM) were injected at the indicated times. *E* and *F*: data on basal and maximum respiratory rates represent means ± SE from 6–8 independent experiments performed on 4 cell populations. ***P < 0.001, Gal- vs. Glu-containing medium. ^b|^*P* < 0.05, ^a|^*P* < 0.01, and ^a′|^*P* < 0.001, nonstimulated vs. nucleotide- or Ado-stimulated cells.

Our data presented in Figs. 1 and 2 strongly indicate that OXPHOS is necessary for VVEC angiogenic responses. Although regulation of mitochondrial OXPHOS via signaling mechanisms and various metabolic substrates has been demonstrated in several cell types, the data on EC are limited. Using Seahorse analyzer, we examined if OXPHOS (measured as OCR) can be regulated by extracellular nucleotides and adenosine. Since galactose was reported to increase oxidative capacity in different cell types (3, 41), we also investigated the possible modulation OXPHOS in VVEC by glucose and galactose. Culturing VVEC in the presence of 20 mM galactose and 5 mM glucose for 7 days increased basal OCR, compared with 25 mM glucose (Fig. 6). Galactose culture condition also resulted in a significant increase in maximal OCR rate (FCCP response, or State 3 uncoupled). Treatment with oligomycin resulted in near equivalent decreases in OCR in cells cultured in both high-galactose and high-glucose medium, indicating near equivalent ATP production via OXPHOS in mitochondria under conditions tested. Treatment with rotenone and antimycin A (complex I and III inhibitors, respectively) resulted in OCR decrease below the oligomycin-treated level, suggesting a negligible oligomycin-insensitive respiration in VVEC under both high-glucose and high-galactose conditions (Fig. 6, *A* and *E*). Stimulation with extracellular ATP (100 μM), MeSADP (100 μM), and to a lesser extend adenosine (100 μM) increased basal OCR in high-glucose conditions, whereas, in high-galactose conditions, ATP was not effective (Fig. 6). In the same experiments in the high-glucose condition, ATP and MeSADP increased maximal OCR in VVEC. In high galactose, ATP and adenosine did not increase maximal OCR in VVEC, and MeSADP had a negligible effect (Fig. 6). Together, these data indicate that activation of purinergic receptors and incubation with galactose increase oxidative capacity of VVEC.

#### Extracellular ATP elevates mitochondrial Ca^2+^ and upregulates mitochondrial metabolic enzymes.

**Fig. 7.**
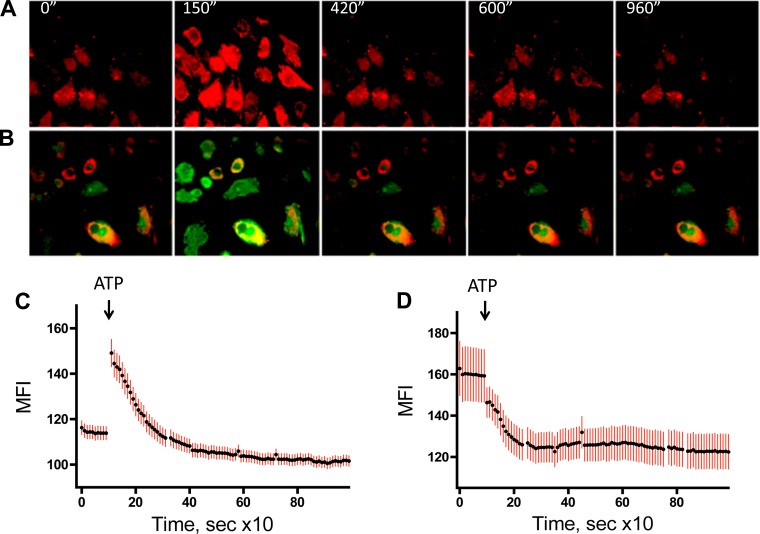
Extracellular ATP increases Ca^2+^ in VVEC mitochondria. *A*: VVEC were cultured on glass-bottom dishes (MatTek, Ashland, MA) and growth-arrested in DMEM without serum for 72 h. For Ca^2+^ measurements, cells were loaded with 1 μM rhodamine 2AM for 30 min at room temperature, washed with media, and left to recover for 10 min to reduce spontaneous intrinsic Ca^2+^ activity. Time-lapse image acquisition was started, and cells were perfused with media containing ATP (100 μM) (rhodamine 2AM in red). *B*: a similar experiment was performed on cells loaded with MitoTracker Deep Red FM to visualize the mitochondria (red) and membrane-permeable Ca^2+^ probe rhodamine 2AM (green). Time-lapse images were captured with 10-s intervals on Nikon TE2000 microscope equipped with Cooke CCD SensiCam. SlideBook software was used for image acquisition and analysis. Averaged single-cell Ca^2+^ time-lapse traces (*n* = 15–20; means ± SE, plotted in green bars) of control (*C*) and 1 μM ruthenium red-treated cells (*D*) are shown for each time point. Shown is 1 representative image from 3 independent experiments. MFI, mean fluorescence intensity.

Elevation of intracellular Ca^2+^ is physiologically important for both the regulation of OXPHOS and cell proliferation (33, 46, 57). Moreover, our laboratory's previous study demonstrated that P2Y_1_- and P2Y_13_-mediated mitogenic signaling in VVEC are Ca^2+^ dependent, but purinergic regulation of mitochondrial Ca^2+^ in VVEC remain unexplored. To assess this possibility, mitochondrial Ca^2+^ responses were determined in VVEC loaded with membrane-permeable Ca^2+^ indicator rhodamine 2AM (Fig. 7). The maximal response was observed at 150 s after stimulation and declined by 420 s (Fig. 7, *A* and *C*). Loading cells with rhodamine 2AM and Mitotracker DeepRed allowed simultaneous visualization of Ca^2+^ signal and mitochondria (Fig. 7*B*). The observed ATP-induced Ca^2+^ increase was attenuated by preincubation (1 μM, 30 min), with a specific inhibitor of Ca^2+^ transport to mitochondria, ruthenium red (Fig. 7*D*), demonstrating mitochondrial localization of the signal.

**Fig. 8.**
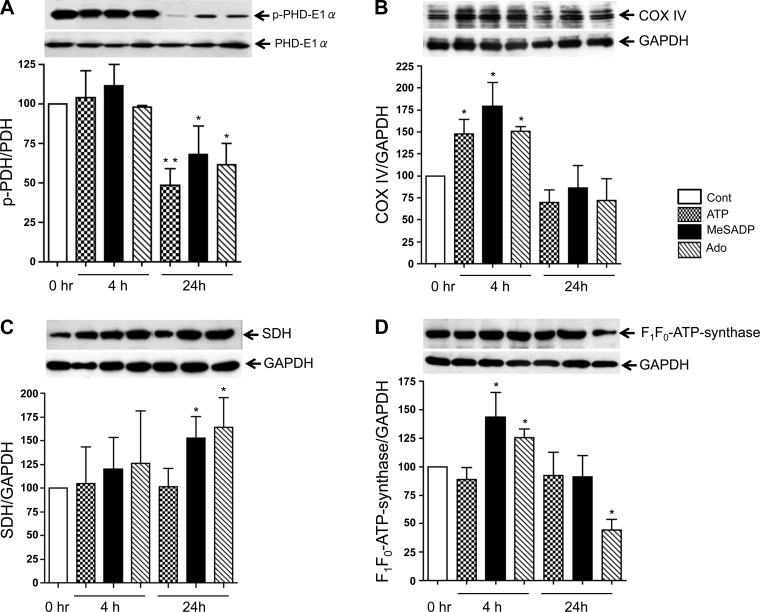
Extracellular ATP, MeSADP, and adenosine (Ado) regulate enzymes involved in OXPHOS in VVEC. Growth-arrested cells were stimulated with ATP, MeSADP, or Ado (100 μM each). Phospho-PDH-E1α (*A*), COX IV (*B*), SDH (*C*), and F_0_F_1_ ATP (*D*) synthase β-subunit were determined in total cell lysates by Western blot analysis. Values are means ± SE from 3–5 independent experiments performed on 3 cell populations. **P < 0.01 and *P < 0.05, nonstimulated vs. agonist-stimulated cells.

As OXPHOS is important for VVEC angiogenesis and is regulated via purinergic receptors, we next examined whether purinergic receptor agonists modulate the expression of mitochondrial enzymes. As shown in Fig. 8*A*, stimulation with extracellular ATP, MeSADP, and adenosine for 24 h decreased phosphorylation of the PDH-E1α, indicating its activation. We also observed significant increase in the expression level of COX IV, after 4 h exposure, to indicated purinergic receptor agonists (Fig. 8*B*). In addition, MeSADP and adenosine, but not ATP, slightly increased the expression of SDH (Fig. 8*C*) and F_1_F_0_ ATP synthase β-subunit (Fig. 8*D*) observed at 24 and 4 h, respectively. In contrast, adenosine downregulated F_1_F_0_ ATP synthase β-subunit expression at 24 h.

#### PI3K and Akt are involved in the regulation of VVEC metabolic pathways.

**Fig. 9.**
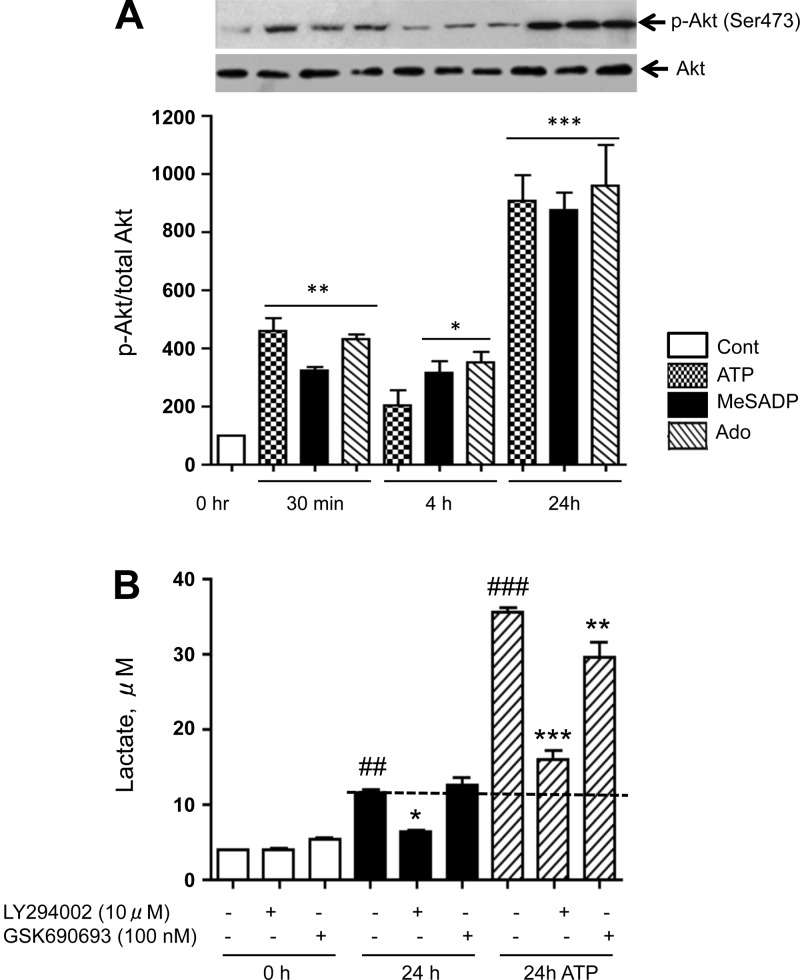
Purinergic receptor-mediated metabolic regulation of VVEC involves the PI3K/Akt pathway. *A*: phospho-Akt and Akt were determined by Western blot analysis in control and ATP-stimulated VVEC, which were either treated or untreated with LY294002 (10 μM) or GSK690693 (100 nM). *B*: lactate production was determined in the conditioned media of control and ATP-stimulated cells that were either untreated or treated with PI3K inhibitor LY294002 (10 μM) or Akt inhibitor GSK690693 (100 nM) for 20 min. Values are means ± SE from 3 independent experiments. ##*P* < 0.01 and ###*P* < 0.01, compared with basal (0 h) condition. *P < 0.05, **P < 0.01, and ***P < 0.01, inhibitor-treated vs. untreated cells. Data are representative of 3 or more independent experiments.

PI3K/Akt pathway plays an important role in cell proliferation, growth factor-induced glycolysis, and mitochondrial biogenesis (16, 78, 81). Therefore, we examined the effects of PI3K and Akt inhibitors on lactate production and the expression of metabolic enzymes. Treatment with extracellular ATP, MeSADP, and adenosine induced a biphasic Akt phosphorylation with an early response observed at 30 min, which was followed up by a more extensive phosphorylation observed at 24 h (Fig. 9*A*). Pretreatment with PI3K inhibitor, LY294002 (10 μM), significantly decreased the basal and ATP-stimulated lactate levels by 44% and 67%, respectively. Pretreatment with an Akt inhibitor, GSK690693 (10 nM), attenuated ATP-stimulated lactate production by ∼17% (Fig. 9*B*), indicating the involvement of Akt and possibly another intracellular kinase pathways, in the regulation of glycolysis in VVEC. We also demonstrated that pretreatment with LY294002 decreased phospho-Akt (Fig. 9*C*), and both LY294002 and GSK690693, although to a different extend, decreased the levels of HK, phospho-PFK3, LDH, SDH, and GLUT-1 in ATP-stimulated cells (Table 1).

**Table 1.** Effect of PI3K and Akt inhibitors on ATP-stimulated metabolic enzyme expression in VVEC

Metabolic Proteins	LY2904002 (10 μM)	GSK690693 (10 nM)
HK	62.5 ± 0.9^*a*^	40.7 ± 9.3^*a*^
p-PFKB3	53.0 ± 10.4^*b*^	76.1 ± 9.9^*c*^
LDH	108 ± 1.0	133.2 ± 21.5^*c*^
GLUT-1	30.1 ± 14.6^*b*^	29.3 ± 12.6^*b*^
SDH	72.5 ± 4.9^*b*^	55.2 ± 2.1^*b*^
p-Akt	48.0 ± 14.1^*c*^	168.2 ± 62.1

Values are means ± SE in % of ATP-stimulated level. Growth-arrested cells (72 h, without serum) were preincubated with LY294002 (10 μM) or GSK690693 (10 nM) for 30 min, or remain untreated and stimulated with 100 μM ATP for 24 h. The expression of indicated metabolic proteins was determined by Western blot analysis, as described in methods. The calculated values from 3–4 independent experiments represent the percentage of indicated protein expression in LY294002- and GSK690693-treated cells compared with the expression in ATP-stimulated cells (taken as 100%).

a *P* < 0.001,

b *P* < 0.01, and

c *P* < 0.05.

## DISCUSSION

Angiogenesis of the VV plays a pathological role in the development of hypoxia-induced PH. In the present study, using VVEC isolated from PA VV, we extended our previous work to evaluate a possible role of cellular energy pathways in VVEC angiogenic responses and regulation of these pathways by purinergic receptors. Our study revealed distinct roles of P2R (ATP and MeSADP) and P1R (adenosine) in VVEC angiogenic and metabolic responses. We found that glycolysis and OXPHOS are both necessary for VVEC angiogenesis, suggesting that VVEC may represent a “bivalent” metabolic phenotype with a greater reliance on glycolysis in response to P2R stimulation. In addition, consistent with a previously reported role of PI3K and Akt pathways in VVEC proliferative responses (30, 80), our study demonstrated a role of the PI3K/Akt signaling pathway in the induction of several key glycolytic and OXPHOS enzymes, as well as the GLUT-1 transporter.

The relationship between cellular metabolism and proliferative responses has been investigated in various cell types. Preference for aerobic glycolysis is considered to be a characteristic feature of tumor cells and other cell types with a highly proliferative potential (44, 72). Increase in proliferative and angiogenic activity is accompanied by utilization of glucose (32), increase of LDH activity (51, 52), and activation of glycolysis (2, 22, 23). In the present study using 2-DG, rotenone, oligomycin, and FCCP, we evaluated a relative contribution of OXPHOS and glycolysis on purinergic receptor-mediated VVEC angiogenesis. Consistent with previous reports showing involvement of the glycolytic pathway to angiogenic potential, we found that treatment with 2-DG attenuates both P1R and P2R agonists-stimulated VVEC DNA synthesis and P2R-stimulated migration. Contrary to our expectations, treatment with 2-DG did not affect nucleotide-mediated tube formation on Matrigel, suggesting that OXPHOS may be relatively more important for tube formation response. Our observations on the role of glycolysis in VVEC angiogenesis are in agreement with recent findings for a critical role of glycolysis in vessel sprouting during retinal angiogenesis (22, 83). Noteworthy, in this model, VEGFR2-increased expression of PFKFB3 leads to localized glycolytic ATP production to lamelopodia and filopodia (23). Our data are also in agreement with the previous studies that demonstrated that PA EC from patients with idiopathic pulmonary arterial hypertension exhibit abnormal metabolic phenotype characterized by increased proliferative responses, which is accompanied by resistance to apoptosis and metabolic switch to glycolysis (82).

**Fig. 10.**
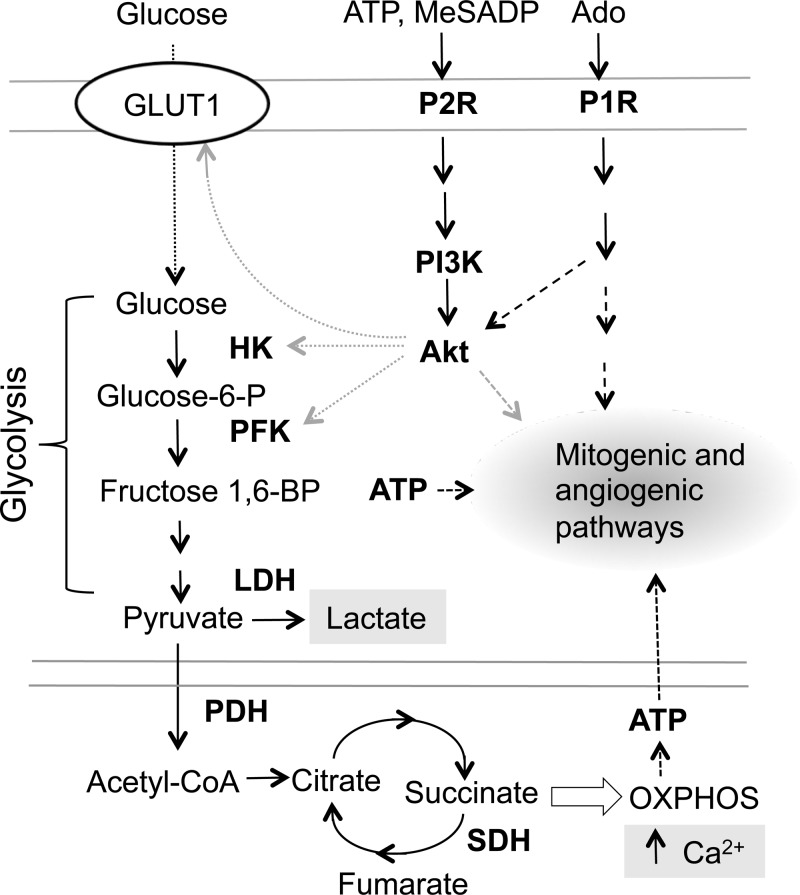
Schematic presentation that demonstrates a link between purinergic receptor-mediated signaling and cellular metabolic pathways. Stimulation of purinergic receptors (presumably P2 subtype and, to a lesser extent, P1 subtype) by extracellular ATP, MeSADP, and adenosine (Ado) results in activation of PI3K/Akt pathway, upregulation of glucose transporter-1 (GLUT-1), as well as metabolic enzymes involved in glycolysis [hexokinase (HK) phosphofructokinase (PFK) B3, and lactate dehydrogenase (LDH)] and oxidative phosphorylation (OXPHOS) [pyruvate dehydrogenase (PDH)-E1α, COX IV, succinate dehydrogenase (SDH), and F_0_F_1_ATP synthase]. As a result, activation of glycolysis and OXPHOS, and increase in mitochondrial Ca^2+^, can be observed in VVEC. P2R, P2 purinergic receptors; P1R, P1 purinergic receptors. Solid arrows indicate functional connection between proteins and pathways, dashed arrows indicate a cross talk between pathways, and dotted arrows indicate transport glucose and ATP and functional connection between Akt and its target proteins.

Regulation of glycolysis through the receptor-dependent mechanisms has been reported for several pro-angiogenic factors, including VEGF, endothelin-1, IL-1β, TNF-α, and ATP (6, 9, 71, 73, 77). Although some studies postulated coordinated regulation of metabolic and signaling activities, regulation on cellular energy pathways by extracellular nucleotides in the context of angiogenesis has not been previously investigated. Here, we present evidence that P2R agonists, ATP and MeSADP, but not P1R agonist, adenosine, upregulate VVEC glycolytic activity. This response was evaluated as increased lactate production, increase in ECAR, and stimulation of PFKB3 and HK2, enzymes that represent the essential control points in glycolysis (Fig. 10). Despite increased lactate production, we did not observe any significant effects of purinergic receptor agonists on LDH expression, suggesting that enzymatic activity, but not the enzyme level, is induced by P2R agonists. In addition, our study demonstrates P2R-mediated upregulation and plasma membrane translocation of the facilitated glucose transporter GLUT-1. In agreement with our findings, others have shown P2R-mediated GLUT-1 and GLUT-4 translocation in C2C12 skeletal muscle cells and 3T3-L1 adipocytes, as well as VEGF-mediated GLUT-1 translocation to the plasma membrane in retinal EC (8, 42, 66). Moreover, transfection of P2X_7_ in human embryonic kidney-293 cells resulted in serum-independent growth, upregulation of glycolytic enzymes, GLUT-1, and increase of mitochondrial Ca^2+^ (1, 6). Therefore, together with previous findings, our study presents evidence for the role of purinergic control of glycolysis coupled to angiogenic activation of VVEC.

The importance of mitochondria and oxidative metabolism has been demonstrated in oncogenic transformation (31, 67), cell differentiation (54), neo-angiogenesis (18), and metabolic adaptation (29, 34, 38, 79), indicating that glycolysis is not exclusive to energy production, especially in slow-growing tumors (reviewed in Ref. 40). Some cancer cells maintain mitochondrial function to rapidly switch from glycolysis to OXPHOS during carcinogenesis (65). Studies on skeletal muscle and retina demonstrate the importance of mitochondrial biogenesis for the angiogenic response mediated by VEGF-1/peroxisome proliferator-activated receptor-α coactivator-1 (PGC-1α) pathway (60, 61). Using rotenone (complex I inhibitor) and oligomycin (an inhibitor of F_1_F_0_ ATP synthase), we demonstrated a critical role of OXPHOS in VVEC angiogenesis. Rotenone and oligomycin were almost equipotent in downregulating DNA synthesis, migration, and tube formation, while rotenone had more dramatic inhibitory effect on DNA synthesis compared with migration. Notably, the inhibitory effects of rotenone and oligomycin indicate that the glycolytic pathway cannot fully maintain VVEC angiogenic capabilities, suggesting mitochondrial activity is necessary to maintain VVEC responses. In all studies, mitochondrial membrane potential (Δψ_m_) uncoupler FCCP had a potent inhibitory effect on VVEC angiogenesis, including tube formation, which is considered a later, differentiation-associated angiogenic response. Consistent with our observation, data from tumor angiogenesis and wound-healing models demonstrate that the mitochondrial uncoupler embelin impairs neo-angiogenesis by selectively targeting proliferation of EC (18). It was also demonstrated that Δψ_m_, the predominant component of an electrochemical gradient, plays an important role in differentiation of neuroblastoma and embryonic stem cells (47, 74), indicating that Δψ_m_ may have a role in regulation of cellular responses. Therefore, despite the common acceptance of EC as a “glycolytic” cell type, our study and those of others (20, 25, 64) prove that mitochondria function is required for angiogenesis and serve to further characterize EC phenotypes from both quiescent and angiogenic vessels.

The data on the role of OXPHOS and its regulation by extracellular signals are in agreement with several previous studies that link proliferative responses and mitochondrial function. For example, PGC-1α-mediated mitochondrial biogenesis is important for VEGF- and exercise-stimulated angiogenic responses in skeletal muscle and retina (7, 17, 61, 81). Fibroblast growth factor 21 was shown to regulate energy metabolism by activating AMP-activated protein kinase-sirtuin 1-PGC-1α in the adipocyte 3T3-L1 line (15). In addition, recent studies demonstrated insulin-dependent regulation of OXPHOS, mitochondrial protein expression, and ATP production in skeletal muscle (10, 11, 70). In line with these observations, our study demonstrates that P2R agonists, ATP and MeSADP (and to a lesser extent, P1R agonist adenosine), induce both basal and maximal OCR and upregulate proteins critically involved in oxidative metabolism, i.e., PDH, SDH, COX IV, and β-subunit of mitochondrial F_1_F_0_ ATP synthase. Furthermore, it was previously shown that OXPHOS can be regulated by substrate availability (37), and that skeletal muscle response to galactose is associated with increased mitochondrial biogenesis (3). We found that culturing VVEC in the presence of 20 mM galactose and low glucose increased basal and purinergic agonist-induced basal and maximal OCR, indicating a shift of cellular metabolism toward a more oxidative one. This effect of extracellular nucleotides was potentiated in cells grown in the presence of galactose, suggesting that mitochondrial function can be regulated metabolically and via receptor-mediated signaling pathways.

Intracellular and mitochondrial Ca^2+^ homeostasis plays an important role in metabolic and proliferative signaling (33, 57). Although mitochondria have emerged as important targets of agonist-dependent Ca^2+^ elevation (33, 35, 39), data on purinergic regulation of mitochondrial Ca^2+^ responses are very limited (85, 86). It was established that increased mitochondrial Ca^2+^ results in increased OXPHOS via the activation of Ca^2+^-sensitive dehydrogenases, leading to accumulation of reduced cofactors (NADH, FADH_2_) necessary for Δψ_m_ and ATP synthesis (33). Our laboratory's previous studies demonstrated a role of P2Y_1_R and P2Y_13_R in the elevation of intracellular Ca^2+^ coupled to the activation of mitogenic signaling pathways in VVEC (46). In the present study, we demonstrated that extracellular ATP (P2YR agonist) elevates mitochondrial Ca^2+^ in VVEC. This observation is consistent with other reports demonstrating an increase in mitochondrial Ca^2+^ in response to purinergic stimulation of astrocytes and Sertoli cells (75, 86). Increase of OCR rate and glucose uptake in response to Ca^2+^ mobilizing hormones has also been demonstrated in hepatocytes and mononuclear cells (43, 45). Taken together, our data support the idea of receptor-dependent regulation of mitochondria and a role of mitochondria in VVEC angiogenic response (Fig. 10).

We previously reported that the PI3K/Akt pathway is critical for ATP-induced angiogenic responses in VVEC (30, 80). Our present study implicates PI3K/Akt pathway in the angiogenic regulation of glucose uptake, and cellular energy metabolism in VVEC. Notably, we found biphasic (around 30 min and 24 h) response of Akt phosphorylation in ATP, MeSADP, and adenosine-treated cells. This response can be explained by the involvement of intracellular network of PI3K, PTEN (phosphatase and tensin homolog), PP1, and mammalian target of rapamycin (mTOR) C2 pathways that regulate time-dependent phosphorylation and dephosphorylation signals (12, 13, 48, 59). Activation of these pathways, as well as an autocrine positive feedback signaling via ATP and cytokine release from VVEC, cannot be excluded and may explain a secondary (24 h) response of Akt phosphorylation in VVEC.

We also demonstrate that P2R-induced lactate production, phosphorylation of PFK3, and upregulation of HK and SDH are all sensitive to PI3K inhibitor LY294002 and, to a lesser extent, to Akt inhibitor, GSK690693, suggesting that PI3K may play a central role in the glycolysis regulation, whereas there might be additional kinase pathways, in addition to Akt, responsible for the regulation of GLUT-1 and/or glycolytic enzymes (Fig. 10). In line with our observations on metabolic involvement of PI3K/Akt pathway, insulin-induced Akt translocation to mitochondria has been shown to stimulate PDH activity and mitochondrial ATP production in cardiomyocytes (24, 84). Akt and mTOR have been demonstrated to regulate mitochondrial activity via the activation Yin-Yang 1 and PGC-1α transcription factors (19, 63), pointing out the importance of the Akt/mTOR pathway for cellular energy pathways.

Collectively, the results from our study present new evidence that VVEC angiogenesis requires functional glycolysis and OXPHOS cellular energy pathways that can be modulated by purinergic receptor activation and metabolic substrates. Given the autocrine/paracrine role of extracellular ATP in hypoxia-induced VV angiogenesis, the results from our study may suggest development of new therapeutic strategies to limit pathological angiogenesis in cardiovascular diseases via simultaneous targeting of purinergic receptors and cellular metabolic pathways.

## GRANTS

The study was supported by National Heart, Lung, and Blood Institute Grants R01-HL-086783 (to E. V. Gerasimovskaya) and PPG-HL-14985 (to K. R. Stenmark).

## DISCLOSURES

No conflicts of interest, financial or otherwise, are declared by the author(s).

## AUTHOR CONTRIBUTIONS

M.L., P.W., D.S., V.K., N.B., T.L., and E.V.G. performed experiments; M.L., P.W., D.S., N.B., T.L., and E.V.G. analyzed data; M.L., D.S., V.K., N.B., T.L., and E.V.G. prepared figures; M.L., P.W., D.S., V.K., T.L., K.R.S., and E.V.G. edited and revised manuscript; M.L., P.W., D.S., V.K., N.B., T.L., P.P., K.R.S., and E.V.G. approved final version of manuscript; V.K., P.P., and E.V.G. interpreted results of experiments; E.V.G. drafted manuscript.

## References

[1] Adinolfi E, Callegari MG, Ferrari D, Bolognesi C, Minelli M, Wieckowski MR, Pinton P, Rizzuto R, Di Virgilio F Basal activation of the P2X7 ATP receptor elevates mitochondrial calcium and potential, increases cellular ATP levels, and promotes serum-independent growth. Mol Biol Cell 16: 3260–3272, 2005. 15901833 10.1091/mbc.E04-11-1025 PMC1165409

[2] Agathocleous M, Love NK, Randlett O, Harris JJ, Liu J, Murray AJ, Harris WA Metabolic differentiation in the embryonic retina. Nat Cell Biol 14: 859–864, 2012. 22750943 10.1038/ncb2531 PMC3442239

[3] Aguer C, Gambarotta D, Mailloux RJ, Moffat C, Dent R, McPherson R, Harper ME Galactose enhances oxidative metabolism and reveals mitochondrial dysfunction in human primary muscle cells. PLoS One 6: e28536, 2011. 22194845 10.1371/journal.pone.0028536 PMC3240634

[4] Aird WC Phenotypic heterogeneity of the endothelium. I. Structure, function, and mechanisms. Circ Res 100: 158–173, 2007. 17272818 10.1161/01.RES.0000255691.76142.4a

[5] Al-Mehdi AB, Pastukh VM, Swiger BM, Reed DJ, Patel MR, Bardwell GC, Pastukh VV, Alexeyev MF, Gillespie MN Perinuclear mitochondrial clustering creates an oxidant-rich nuclear domain required for hypoxia-induced transcription. Sci Signal 5: ra47, 2012. 22763339 10.1126/scisignal.2002712 PMC3565837

[6] Amoroso F, Falzoni S, Adinolfi E, Ferrari D, Di Virgilio F The P2X7 receptor is a key modulator of aerobic glycolysis. Cell Death Dis 3: e370, 2012. 22898868 10.1038/cddis.2012.105 PMC3434661

[7] Arany Z, Foo SY, Ma Y, Ruas JL, Bommi-Reddy A, Girnun G, Cooper M, Laznik D, Chinsomboon J, Rangwala SM, Baek KH, Rosenzweig A, Spiegelman BM HIF-independent regulation of VEGF and angiogenesis by the transcriptional coactivator PGC-1alpha. Nature 451: 1008–1012, 2008. 18288196 10.1038/nature06613

[8] Balasubramanian R, Robaye B, Boeynaems JM, Jacobson KA Enhancement of glucose uptake in mouse skeletal muscle cells and adipocytes by P2Y6 receptor agonists. PLoS One 9: e116203, 2014. 25549240 10.1371/journal.pone.0116203 PMC4280206

[9] Ben-Shlomo I, Kol S, Roeder LM, Resnick CE, Hurwitz A, Payne DW, Adashi EY Interleukin (IL)-1beta increases glucose uptake and induces glycolysis in aerobically cultured rat ovarian cells: evidence that IL-1beta may mediate the gonadotropin-induced midcycle metabolic shift. Endocrinology 138: 2680–2688, 1997. 9202204 10.1210/endo.138.7.5229

[10] Boirie Y Insulin regulation of mitochondrial proteins and oxidative phosphorylation in human muscle. Trends Endocrinol Metab 14: 393–394, 2003. 14580754 10.1016/j.tem.2003.09.002

[11] Boirie Y, Short KR, Ahlman B, Charlton M, Nair KS Tissue-specific regulation of mitochondrial and cytoplasmic protein synthesis rates by insulin. Diabetes 50: 2652–2658, 2001. 11723046 10.2337/diabetes.50.12.2652

[12] Camire RB, Beaulac HJ, Brule SA, McGregor AI, Lauria EE, Willis CL Biphasic modulation of paracellular claudin-5 expression in mouse brain endothelial cells is mediated through the phosphoinositide-3-kinase/AKT pathway. J Pharmacol Exp Ther 351: 654–662, 2014. 25281324 10.1124/jpet.114.218339 PMC4244583

[13] Cantoni S, Galletti M, Zambelli F, Valente S, Ponti F, Tassinari R, Pasquinelli G, Galie N, Ventura C Sodium butyrate inhibits platelet-derived growth factor-induced proliferation and migration in pulmonary artery smooth muscle cells through Akt inhibition. FEBS J 280: 2042–2055, 2013. 23463962 10.1111/febs.12227

[14] Carmeliet P Angiogenesis in health and disease. Nat Med 9: 653–660, 2003. 12778163 10.1038/nm0603-653

[15] Chau MD, Gao J, Yang Q, Wu Z, Gromada J Fibroblast growth factor 21 regulates energy metabolism by activating the AMPK-SIRT1-PGC-1alpha pathway. Proc Natl Acad Sci U S A 107: 12553–12558, 2010. 20616029 10.1073/pnas.1006962107 PMC2906565

[16] Chen J, Somanath PR, Razorenova O, Chen WS, Hay N, Bornstein P, Byzova TV Akt1 regulates pathological angiogenesis, vascular maturation and permeability in vivo. Nat Med 11: 1188–1196, 2005. 16227992 10.1038/nm1307 PMC2277080

[17] Chinsomboon J, Ruas J, Gupta RK, Thom R, Shoag J, Rowe GC, Sawada N, Raghuram S, Arany Z The transcriptional coactivator PGC-1alpha mediates exercise-induced angiogenesis in skeletal muscle. Proc Natl Acad Sci U S A 106: 21401–21406, 2009. 19966219 10.1073/pnas.0909131106 PMC2795492

[18] Coutelle O, Hornig-Do HT, Witt A, Andree M, Schiffmann LM, Piekarek M, Brinkmann K, Seeger JM, Liwschitz M, Miwa S, Hallek M, Kronke M, Trifunovic A, Eming SA, Wiesner RJ, Hacker UT, Kashkar H Embelin inhibits endothelial mitochondrial respiration and impairs neoangiogenesis during tumor growth and wound healing. EMBO Mol Med 6: 624–639, 2014. 24648500 10.1002/emmm.201303016 PMC4023885

[19] Cunningham JT, Rodgers JT, Arlow DH, Vazquez F, Mootha VK, Puigserver P mTOR controls mitochondrial oxidative function through a YY1-PGC-1alpha transcriptional complex. Nature 450: 736–740, 2007. 18046414 10.1038/nature06322

[20] Davidson SM, Duchen MR Endothelial mitochondria: contributing to vascular function and disease. Circ Res 100: 1128–1141, 2007. 17463328 10.1161/01.RES.0000261970.18328.1d

[21] Davie NJ, Crossno JT Jr, Frid MG, Hofmeister SE, Reeves JT, Hyde DM, Carpenter TC, Brunetti JA, McNiece IK, Stenmark KR Hypoxia-induced pulmonary artery adventitial remodeling and neovascularization: contribution of progenitor cells. Am J Physiol Lung Cell Mol Physiol 286: L668–L678, 2004. 12754186 10.1152/ajplung.00108.2003

[22] De Bock K, Georgiadou M, Carmeliet P Role of endothelial cell metabolism in vessel sprouting. Cell Metab 18: 634–647, 2013. 23973331 10.1016/j.cmet.2013.08.001

[23] De Bock K, Georgiadou M, Schoors S, Kuchnio A, Wong BW, Cantelmo AR, Quaegebeur A, Ghesquiere B, Cauwenberghs S, Eelen G, Phng LK, Betz I, Tembuyser B, Brepoels K, Welti J, Geudens I, Segura I, Cruys B, Bifari F, Decimo I, Blanco R, Wyns S, Vangindertael J, Rocha S, Collins RT, Munck S, Daelemans D, Imamura H, Devlieger R, Rider M, Van Veldhoven PP, Schuit F, Bartrons R, Hofkens J, Fraisl P, Telang S, Deberardinis RJ, Schoonjans L, Vinckier S, Chesney J, Gerhardt H, Dewerchin M, Carmeliet P Role of PFKFB3-driven glycolysis in vessel sprouting. Cell 154: 651–663, 2013. 23911327 10.1016/j.cell.2013.06.037

[24] Deng W, Leu HB, Chen Y, Chen YH, Epperson CM, Juang C, Wang PH Protein kinase B (PKB/AKT1) formed signaling complexes with mitochondrial proteins and prevented glycolytic energy dysfunction in cultured cardiomyocytes during ischemia-reperfusion injury. Endocrinology 155: 1618–1628, 2014. 24601882 10.1210/en.2013-1817 PMC3990846

[25] Dranka BP, Hill BG, Darley-Usmar VM Mitochondrial reserve capacity in endothelial cells: the impact of nitric oxide and reactive oxygen species. Free Radic Biol Med 48: 905–914, 2010. 20093177 10.1016/j.freeradbiomed.2010.01.015 PMC2860730

[26] Dromparis P, Sutendra G, Michelakis ED The role of mitochondria in pulmonary vascular remodeling. J Mol Med (Berl) 88: 1003–1010, 2010. 20734021 10.1007/s00109-010-0670-x

[27] Eelen G, de Zeeuw P, Simons M, Carmeliet P Endothelial cell metabolism in normal and diseased vasculature. Circ Res 116: 1231–1244, 2015. 25814684 10.1161/CIRCRESAHA.116.302855 PMC4380230

[28] Elstrom RL, Bauer DE, Buzzai M, Karnauskas R, Harris MH, Plas DR, Zhuang H, Cinalli RM, Alavi A, Rudin CM, Thompson CB Akt stimulates aerobic glycolysis in cancer cells. Cancer Res 64: 3892–3899, 2004. 15172999 10.1158/0008-5472.CAN-03-2904

[29] Funes JM, Quintero M, Henderson S, Martinez D, Qureshi U, Westwood C, Clements MO, Bourboulia D, Pedley RB, Moncada S, Boshoff C Transformation of human mesenchymal stem cells increases their dependency on oxidative phosphorylation for energy production. Proc Natl Acad Sci U S A 104: 6223–6228, 2007. 17384149 10.1073/pnas.0700690104 PMC1851087

[30] Gerasimovskaya EV, Woodward HN, Tucker DA, Stenmark KR Extracellular ATP is a pro-angiogenic factor for pulmonary artery vasa vasorum endothelial cells. Angiogenesis 11: 169–182, 2008. 18071915 10.1007/s10456-007-9087-8 PMC2480488

[31] Gough DJ, Corlett A, Schlessinger K, Wegrzyn J, Larner AC, Levy DE Mitochondrial STAT3 supports Ras-dependent oncogenic transformation. Science 324: 1713–1716, 2009. 19556508 10.1126/science.1171721 PMC2840701

[32] Greiner EF, Guppy M, Brand K Glucose is essential for proliferation and the glycolytic enzyme induction that provokes a transition to glycolytic energy production. J Biol Chem 269: 31484–31490, 1994. 7989314

[33] Griffiths EJ, Rutter GA Mitochondrial calcium as a key regulator of mitochondrial ATP production in mammalian cells. Biochim Biophys Acta 1787: 1324–1333, 2009. 19366607 10.1016/j.bbabio.2009.01.019

[34] Guppy M, Leedman P, Zu X, Russell V Contribution by different fuels and metabolic pathways to the total ATP turnover of proliferating MCF-7 breast cancer cells. Biochem J 364: 309–315, 2002. 11988105 10.1042/bj3640309 PMC1222574

[35] Hajnoczky G, Csordas G, Krishnamurthy R, Szalai G Mitochondrial calcium signaling driven by the IP3 receptor. J Bioenerg Biomembr 32: 15–25, 2000. 11768758 10.1023/a:1005504210587

[36] Hanahan D, Weinberg RA Hallmarks of cancer: the next generation. Cell 144: 646–674, 2011. 21376230 10.1016/j.cell.2011.02.013

[37] Harjes U, Bensaad K, Harris AL Endothelial cell metabolism and implications for cancer therapy. Br J Cancer 107: 1207–1212, 2012. 23047591 10.1038/bjc.2012.398 PMC3494441

[38] Hooda J, Cadinu D, Alam MM, Shah A, Cao TM, Sullivan LA, Brekken R, Zhang L Enhanced heme function and mitochondrial respiration promote the progression of lung cancer cells. PLoS One 8: e63402, 2013. 23704904 10.1371/journal.pone.0063402 PMC3660535

[39] Jean-Quartier C, Bondarenko AI, Alam MR, Trenker M, Waldeck-Weiermair M, Malli R, Graier WF Studying mitochondrial Ca(2+) uptake–a revisit. Mol Cell Endocrinol 353: 114–127, 2012. 22100614 10.1016/j.mce.2011.10.033 PMC3334272

[40] Jose C, Bellance N, Rossignol R Choosing between glycolysis and oxidative phosphorylation: a tumor's dilemma? Biochim Biophys Acta 1807: 552–561, 2011. 20955683 10.1016/j.bbabio.2010.10.012

[41] Kase ET, Nikolic N, Bakke SS, Bogen KK, Aas V, Thoresen GH, Rustan AC Remodeling of oxidative energy metabolism by galactose improves glucose handling and metabolic switching in human skeletal muscle cells. PLoS One 8: e59972, 2013. 23560061 10.1371/journal.pone.0059972 PMC3613401

[42] Kim MS, Lee J, Ha J, Kim SS, Kong Y, Cho YH, Baik HH, Kang I ATP stimulates glucose transport through activation of P2 purinergic receptors in C(2)C(12) skeletal muscle cells. Arch Biochem Biophys 401: 205–214, 2002. 12054471 10.1016/S0003-9861(02)00056-5

[43] Korzeniewski B, Harper ME, Brand MD Proportional activation coefficients during stimulation of oxidative phosphorylation by lactate and pyruvate or by vasopressin. Biochim Biophys Acta 1229: 315–322, 1995. 7748883 10.1016/0005-2728(95)00008-7

[44] Krejci A Metabolic sensors and their interplay with cell signalling and transcription. Biochem Soc Trans 40: 311–323, 2012. 22435805 10.1042/BST20110767

[45] Kvetny J, Matzen LE Thyroid hormone induced oxygen consumption and glucose-uptake in human mononuclear cells. Thyroidology 1: 5–9, 1989. 2484909

[46] Lyubchenko T, Woodward H, Veo KD, Burns N, Nijmeh H, Liubchenko GA, Stenmark KR, Gerasimovskaya EV P2Y1 and P2Y13 purinergic receptors mediate Ca^2+^ signaling and proliferative responses in pulmonary artery vasa vasorum endothelial cells. Am J Physiol Cell Physiol 300: C266–C275, 2011. 20962269 10.1152/ajpcell.00237.2010 PMC3043637

[47] Mandal S, Lindgren AG, Srivastava AS, Clark AT, Banerjee U Mitochondrial function controls proliferation and early differentiation potential of embryonic stem cells. Stem Cells 29: 486–495, 2011. 21425411 10.1002/stem.590 PMC4374603

[48] Marino M, Acconcia F, Trentalance A Biphasic estradiol-induced AKT phosphorylation is modulated by PTEN via MAP kinase in HepG2 cells. Mol Biol Cell 14: 2583–2591, 2003. 12808053 10.1091/mbc.E02-09-0621 PMC194905

[49] Montani D, Perros F, Gambaryan N, Girerd B, Dorfmuller P, Price LC, Huertas A, Hammad H, Lambrecht B, Simonneau G, Launay JM, Cohen-Kaminsky S, Humbert M C-kit-positive cells accumulate in remodeled vessels of idiopathic pulmonary arterial hypertension. Am J Respir Crit Care Med 184: 116–123, 2011. 21471108 10.1164/rccm.201006-0905OC

[50] Mulligan-Kehoe MJ, Simons M Vasa vasorum in normal and diseased arteries. Circulation 129: 2557–2566, 2014. 24934463 10.1161/CIRCULATIONAHA.113.007189

[51] Parra-Bonilla G, Alvarez DF, Al-Mehdi AB, Alexeyev M, Stevens T Critical role for lactate dehydrogenase A in aerobic glycolysis that sustains pulmonary microvascular endothelial cell proliferation. Am J Physiol Lung Cell Mol Physiol 299: L513–L522, 2010. 20675437 10.1152/ajplung.00274.2009 PMC2957419

[52] Parra-Bonilla G, Alvarez DF, Alexeyev M, Vasauskas A, Stevens T Lactate dehydrogenase a expression is necessary to sustain rapid angiogenesis of pulmonary microvascular endothelium. PLoS One 8: e75984, 2013. 24086675 10.1371/journal.pone.0075984 PMC3784391

[53] Pearce EL Metabolism in T cell activation and differentiation. Curr Opin Immunol 22: 314–320, 2010. 20189791 10.1016/j.coi.2010.01.018 PMC4486663

[54] Pereira SL, Graos M, Rodrigues AS, Anjo SI, Carvalho RA, Oliveira PJ, Arenas E, Ramalho-Santos J Inhibition of mitochondrial complex III blocks neuronal differentiation and maintains embryonic stem cell pluripotency. PLoS One 8: e82095, 2013. 24312632 10.1371/journal.pone.0082095 PMC3847032

[55] Polet F, Feron O Endothelial cell metabolism and tumour angiogenesis: glucose and glutamine as essential fuels and lactate as the driving force. J Intern Med 273: 156–165, 2013. 23216817 10.1111/joim.12016

[56] Prigione A, Adjaye J Modulation of mitochondrial biogenesis and bioenergetic metabolism upon in vitro and in vivo differentiation of human ES and iPS cells. Int J Dev Biol 54: 1729–1741, 2010. 21305470 10.1387/ijdb.103198ap

[57] Rimessi A, Patergnani S, Bonora M, Wieckowski MR, Pinton P Mitochondrial Ca^2+^ remodeling is a prime factor in oncogenic behavior. Front Oncol 5: 143, 2015. 26161362 10.3389/fonc.2015.00143 PMC4479728

[58] Ritman EL, Lerman A The dynamic vasa vasorum. Cardiovasc Res 75: 649–658, 2007. 17631284 10.1016/j.cardiores.2007.06.020 PMC2121590

[59] Rodrik-Outmezguine VS, Chandarlapaty S, Pagano NC, Poulikakos PI, Scaltriti M, Moskatel E, Baselga J, Guichard S, Rosen N mTOR kinase inhibition causes feedback-dependent biphasic regulation of AKT signaling. Cancer Discov 1: 248–259, 2011. 22140653 10.1158/2159-8290.CD-11-0085 PMC3227125

[60] Rowe GC, Jang C, Patten IS, Arany Z PGC-1beta regulates angiogenesis in skeletal muscle. Am J Physiol Endocrinol Metab 301: E155–E163, 2011. 21364124 10.1152/ajpendo.00681.2010 PMC3275155

[61] Saint-Geniez M, Jiang A, Abend S, Liu L, Sweigard H, Connor KM, Arany Z PGC-1alpha regulates normal and pathological angiogenesis in the retina. Am J Pathol 182: 255–265, 2013. 23141926 10.1016/j.ajpath.2012.09.003 PMC3538026

[62] San Martin N, Cervera AM, Cordova C, Covarello D, McCreath KJ, Galvez BG Mitochondria determine the differentiation potential of cardiac mesoangioblasts. Stem Cells 29: 1064–1074, 2011. 21544900 10.1002/stem.654

[63] Schieke SM, Phillips D, McCoy JP Jr, Aponte AM, Shen RF, Balaban RS, Finkel T The mammalian target of rapamycin (mTOR) pathway regulates mitochondrial oxygen consumption and oxidative capacity. J Biol Chem 281: 27643–27652, 2006. 16847060 10.1074/jbc.M603536200

[64] Schleicher M, Shepherd BR, Suarez Y, Fernandez-Hernando C, Yu J, Pan Y, Acevedo LM, Shadel GS, Sessa WC Prohibitin-1 maintains the angiogenic capacity of endothelial cells by regulating mitochondrial function and senescence. J Cell Biol 180: 101–112, 2008. 18195103 10.1083/jcb.200706072 PMC2213620

[65] Smolkova K, Plecita-Hlavata L, Bellance N, Benard G, Rossignol R, Jezek P Waves of gene regulation suppress and then restore oxidative phosphorylation in cancer cells. Int J Biochem Cell Biol 43: 950–968, 2011. 20460169 10.1016/j.biocel.2010.05.003

[66] Sone H, Deo BK, Kumagai AK Enhancement of glucose transport by vascular endothelial growth factor in retinal endothelial cells. Invest Ophthalmol Vis Sci 41: 1876–1884, 2000. 10845612

[67] Sonveaux P, Vegran F, Schroeder T, Wergin MC, Verrax J, Rabbani ZN, De Saedeleer CJ, Kennedy KM, Diepart C, Jordan BF, Kelley MJ, Gallez B, Wahl ML, Feron O, Dewhirst MW Targeting lactate-fueled respiration selectively kills hypoxic tumor cells in mice. J Clin Invest 118: 3930–3942, 2008. 19033663 10.1172/JCI36843 PMC2582933

[68] Spitkovsky D, Sasse P, Kolossov E, Bottinger C, Fleischmann BK, Hescheler J, Wiesner RJ Activity of complex III of the mitochondrial electron transport chain is essential for early heart muscle cell differentiation. FASEB J 18: 1300–1302, 2004. 15180963 10.1096/fj.03-0520fje

[69] Stenmark KR, Yeager ME, El Kasmi KC, Nozik-Grayck E, Gerasimovskaya EV, Li M, Riddle SR, Frid MG The adventitia: essential regulator of vascular wall structure and function. Annu Rev Physiol 75: 23–47, 2013. 23216413 10.1146/annurev-physiol-030212-183802 PMC3762248

[70] Stump CS, Short KR, Bigelow ML, Schimke JM, Nair KS Effect of insulin on human skeletal muscle mitochondrial ATP production, protein synthesis, and mRNA transcripts. Proc Natl Acad Sci U S A 100: 7996–8001, 2003. 12808136 10.1073/pnas.1332551100 PMC164701

[71] Sun X, Kumar S, Sharma S, Aggarwal S, Lu Q, Gross C, Rafikova O, Lee SG, Dasarathy S, Hou Y, Meadows ML, Han W, Su Y, Fineman JR, Black SM Endothelin-1 induces a glycolytic switch in pulmonary arterial endothelial cells via the mitochondrial translocation of endothelial nitric oxide synthase. Am J Respir Cell Mol Biol 50: 1084–1095, 2014. 24392990 10.1165/rcmb.2013-0187OC PMC4068912

[72] Vander Heiden MG, Cantley LC, Thompson CB Understanding the Warburg effect: the metabolic requirements of cell proliferation. Science 324: 1029–1033, 2009. 19460998 10.1126/science.1160809 PMC2849637

[73] Vaughan RA, Garcia-Smith R, Trujillo KA, Bisoffi M Tumor necrosis factor alpha increases aerobic glycolysis and reduces oxidative metabolism in prostate epithelial cells. Prostate 73: 1538–1546, 2013. 23818177 10.1002/pros.22703

[74] Vayssiere JL, Cordeau-Lossouarn L, Larcher JC, Basseville M, Gros F, Croizat B Participation of the mitochondrial genome in the differentiation of neuroblastoma cells. In Vitro Cell Dev Biol 28A: 763–772, 1992. 1483966 10.1007/BF02631065

[75] Veitinger S, Veitinger T, Cainarca S, Fluegge D, Engelhardt CH, Lohmer S, Hatt H, Corazza S, Spehr J, Neuhaus EM, Spehr M Purinergic signalling mobilizes mitochondrial Ca^2+^ in mouse Sertoli cells. J Physiol 589: 5033–5055, 2011. 21859825 10.1113/jphysiol.2011.216309 PMC3225664

[76] Verdegem D, Moens S, Stapor P, Carmeliet P Endothelial cell metabolism: parallels and divergences with cancer cell metabolism. Cancer Metab 2: 19, 2014. 25250177 10.1186/2049-3002-2-19 PMC4171726

[77] Vizan P, Sanchez-Tena S, Alcarraz-Vizan G, Soler M, Messeguer R, Pujol MD, Lee WN, Cascante M Characterization of the metabolic changes underlying growth factor angiogenic activation: identification of new potential therapeutic targets. Carcinogenesis 30: 946–952, 2009. 19369582 10.1093/carcin/bgp083

[78] Ward PS, Thompson CB Signaling in control of cell growth and metabolism. Cold Spring Harb Perspect Biol 4: a006783, 2012. 22687276 10.1101/cshperspect.a006783 PMC3385956

[79] Whitaker-Menezes D, Martinez-Outschoorn UE, Flomenberg N, Birbe RC, Witkiewicz AK, Howell A, Pavlides S, Tsirigos A, Ertel A, Pestell RG, Broda P, Minetti C, Lisanti MP, Sotgia F Hyperactivation of oxidative mitochondrial metabolism in epithelial cancer cells in situ: visualizing the therapeutic effects of metformin in tumor tissue. Cell Cycle 10: 4047–4064, 2011. 22134189 10.4161/cc.10.23.18151 PMC3272287

[80] Woodward HN, Anwar A, Riddle S, Taraseviciene-Stewart L, Fragoso M, Stenmark KR, Gerasimovskaya EV PI3K, Rho, and ROCK play a key role in hypoxia-induced ATP release and ATP-stimulated angiogenic responses in pulmonary artery vasa vasorum endothelial cells. Am J Physiol Lung Cell Mol Physiol 297: L954–L964, 2009. 19684203 10.1152/ajplung.00038.2009 PMC2777489

[81] Wright GL, Maroulakou IG, Eldridge J, Liby TL, Sridharan V, Tsichlis PN, Muise-Helmericks RC VEGF stimulation of mitochondrial biogenesis: requirement of AKT3 kinase. FASEB J 22: 3264–3275, 2008. 18524868 10.1096/fj.08-106468 PMC2518259

[82] Xu W, Koeck T, Lara AR, Neumann D, DiFilippo FP, Koo M, Janocha AJ, Masri FA, Arroliga AC, Jennings C, Dweik RA, Tuder RM, Stuehr DJ, Erzurum SC Alterations of cellular bioenergetics in pulmonary artery endothelial cells. Proc Natl Acad Sci U S A 104: 1342–1347, 2007. 17227868 10.1073/pnas.0605080104 PMC1783136

[83] Xu Y, An X, Guo X, Habtetsion TG, Wang Y, Xu X, Kandala S, Li Q, Li H, Zhang C, Caldwell RB, Fulton DJ, Su Y, Hoda MN, Zhou G, Wu C, Huo Y Endothelial PFKFB3 plays a critical role in angiogenesis. Arterioscler Thromb Vasc Biol 34: 1231–1239, 2014. 24700124 10.1161/ATVBAHA.113.303041 PMC4120754

[84] Yang JY, Yeh HY, Lin K, Wang PH Insulin stimulates Akt translocation to mitochondria: implications on dysregulation of mitochondrial oxidative phosphorylation in diabetic myocardium. J Mol Cell Cardiol 46: 919–926, 2009. 19249309 10.1016/j.yjmcc.2009.02.015 PMC3872534

[85] Yitzhaki S, Hochhauser E, Porat E, Shainberg A Uridine-5′-triphosphate (UTP) maintains cardiac mitochondrial function following chemical and hypoxic stress. J Mol Cell Cardiol 43: 653–662, 2007. 17880998 10.1016/j.yjmcc.2007.07.060

[86] Zheng W, Watts LT, Holstein DM, Prajapati SI, Keller C, Grass EH, Walter CA, Lechleiter JD Purinergic receptor stimulation reduces cytotoxic edema and brain infarcts in mouse induced by photothrombosis by energizing glial mitochondria. PLoS One 5: e14401, 2010. 21203502 10.1371/journal.pone.0014401 PMC3008710

